# On the Issue of Developing Creative Players in Team Sports: A Systematic Review and Critique From a Functional Perspective

**DOI:** 10.3389/fpsyg.2020.575475

**Published:** 2020-10-29

**Authors:** Stephan Zahno, Ernst-Joachim Hossner

**Affiliations:** Movement and Exercise Science, Institute of Sport Science, University of Bern, Bern, Switzerland

**Keywords:** creativity, divergent thinking, invasion games, team sports, motor skill, sensorimotor learning, complex motor skill learning

## Abstract

Driven by the practical goal of developing creative players, several approaches to training creativity have been proposed and underpinned by empirical studies in sport science. However, the scope of these studies encompasses various aspects, which have all been subsumed under the singular label of “creativity.” Therefore, this systematic review aims to disentangle the pursued lines of thought in order to facilitate the derivation of well-grounded recommendations for sports practice. To this end, 38 studies are presented and characterized in terms of their underlying conceptualizations and measures of creativity. In most studies, creativity is conceptualized as a player's domain-specific divergent thinking (DT) ability, reflected by individual differences in the number, variety and originality of ideas he or she is able to generate in response to game situations. Empirical studies indicate that DT can be improved by practice. However, the critical assumption that an enhanced DT ability transfers to creative on-field actions has yet to be tested. On the basis of the reviewed literature, an alternative point of view is proposed. In line with a relational understanding of creativity and a functional approach to behavioral control, it is hypothesized that an enhanced repertoire of sensorimotor skills increases the probability for performing functional solutions that, within a specific social and cultural frame of reference, go beyond the expected and consequently appear creative to the observer. In the context of sports practice, the proposed conceptual re-orientation would then suggest, rather than seeking ways to improve players' DT ability, to target sensorimotor skills that allow players to perform a variety of task-solutions and thus to act less predictably to the opponent—or in other words, more creative.

## Introduction

In team sports, developing creative players is a highly discussed issue (e.g., Wein, 2007; Glynn, 2013). The demand for creativity has been emphasized in many training manuals, such as the FIFA guidelines that state: “creativity must remain at the nucleus of youth development” (Bénézet and Hasler, 2018, p. 10). This practical relevance in turn has attracted great interest to the concept of creativity in sport science over the recent years (De Sa Fardilha and Allen, 2019), where creativity has been described as a “critical attribute” of team sports players (Memmert and Roca, 2019, p. 203) or a “key to expert performance” (Roca et al., 2018, p. 1). As a consequence, research has sought to reveal ways to improve this desirable attribute (Memmert, 2015b). In terms of its function, creativity has been linked to being less predictable to the opponent in game situations. Creative actions that are hard to anticipate are generally considered a decisive element in team sport games and it is further argued that its importance is increasing, especially at elite level, with growing possibilities of collecting information from teams and players' behaviors (Memmert and Roca, 2019).

In a recent review on this topic, De Sa Fardilha and Allen (2019) highlighted the lack of a clear-cut definition of creativity in sports that could be “universally accepted” (p. 17). However, when zooming out to the larger field of creativity research in psychology (e.g., Hennessey and Amabile, 2010), this lack of uniformity does not come as a surprise. Here too, it can be asserted that there exists not only an “abundance of definitions” (Plucker and Makel, 2010, p. 48), but also a “multitude of theoretical perspectives, with different assumptions and methods, and operating at different levels of analysis” (Kozbelt et al., 2010, p. 20). It seems that, as a “multifaceted construct” (Kaufman et al., 2019, p. 732), creativity can only be conceived as an umbrella concept; or as concluded by Dresler (2008), as an “open concept” that provides a superordinate framework encompassing diverse and vaguely overlapping conceptualizations that, in turn, can be applied to various contexts. In an early effort to streamline these varying approaches, Rhodes (1961) proposed the “four Ps” model (often also referred to as “four P's” in the relevant literature). From this perspective, conceptualizations of creativity can be characterized by their – not always mutually exclusive – focus on the creative person (i.e., on creativity as a characteristic of individuals), process (i.e., on the process leading to novel solutions or the process of discovering and creating itself), product (i.e., on a manifest product that is recognized as novel and functional), or press (i.e., on environmental aspects stimulating or restraining creativity). These four perspectives on creativity have become established as “the most often-used structure” (Runco, 2004, p. 661) to organize studies in creativity research.

To draw a further parallel to creativity research in sport science, the study of creativity in general psychology is similarly driven by practical demands. Guilford's (1950) presidential address to the American Psychology Association (APA) is typically referred to as the formal start of scientific research on creativity. In this influential speech, Guilford emphasized the importance of constructing tests to measure individual differences in creativity, defined as a person's more or less developed ability. Guilford's subsequent work (e.g., Guilford, 1967) is highly cited for rooting the concept of divergent thinking (DT), which has been accepted as the mainstream concept for creativity-tests, such as in the widely used Torrance Test of Creative Thinking (TTCT; Torrance, 1966; for an overview on creativity assessments, see Kaufman et al., 2008). DT tasks are open-ended and, as opposed to convergent thinking tasks that require a single solution, prompt one to name all solutions thought possible in response to a posed problem. To identify individual differences from these responses, measures of fluency (number of responses), flexibility (number of different categories of responses) and originality (uniqueness of responses) are commonly quantified to capture DT abilities (Kaufman et al., 2008; Reiter-Palmon et al., 2019). However, recognizing DT as a crucial aspect in the study of creativity certainly does not imply that DT can be equated to creativity. As stated by Runco (2010, p. 413), “There are misunderstandings, the most notable that tests of DT measure creativity, which they do not.” Consequently, harsh criticisms on using DT-tests as creativity-tests can be found in scientific literature (e.g., Dietrich, 2007; Baer and McKool, 2009).

Moreover, the conceptualization of creativity as an individual's ability “to produce work that is both novel (i.e., original, unexpected) and appropriate (i.e., useful, adaptive concerning task constraints)” (Sternberg and Lubart, 1999) has been severely debated in general psychology. A main controversy lies in its generalizability across domains. Therefore, theoretical arguments have been set forth that range from domain-general (Plucker, 1998) and moderately domain-specific (Sternberg and Lubart, 1992) to pronouncedly domain-specific (Baer, 1993, 1998, 2012) (for an overview, see Simonton, 2007; Baer, 2010). In this respect, as an alternative to the conceptualization of creativity as an individual's ability, Westmeyer (1998) proposes a relational concept of creativity. Referring to the four Ps, the product is emphasized rather than the person; that is, in the context of team sports, a specific action in a game situation is regarded as creative rather than the player him/herself possessing an underlying creative ability.

In this alternative view, the existence of a creative product is thus a necessary condition for attributing creativity to a person or a process. The question then arises: what makes a product creative? The answer might be more straightforward than expected at first glance. According to Westmeyer (1998), the product needs to be *evaluated* as creative. In this sense, no product is creative as such; creativity is rather ascribed to the product, relative to its social context, by experts in the respective domain. In other words, creativity is constructed from the relation between the product within a specific context and the individual judging it. This notion perfectly aligns with Csikszentmihalyi's (1999) systems perspective, which highlights the impossibility of separating creativity form persuasion, Gardner's (1993) position that creativity “is inherently a communal or cultural judgement” (p. 36; see also Amabile, 1983) as well as Sternberg's (2019) argument that the evaluation of creativity is always “local with respect to time and place” (p. 403). Thus, creativity would ultimately be attributed to a person who comes up with a product that was judged as creative. This might be, for instance, an unexpected pass with the outside of the foot that de-stabilizes the opponent and instantly creates a goal scoring opportunity. However, within this relational concept, creativity can no longer be conceived as an ability possessed by a person; instead, more differentiated, the focus shifts toward the potential resources for producing creative solutions.

Practically speaking, the discussed conceptual issues inherent to the notion of creativity pose additional challenges for developing well-founded programmes for creativity training in team sports. In this respect, De Sa Fardilha and Allen (2019) conclude that existing programmes—such as the Tactical Creativity Approach (6 D's; Memmert, 2015b), the Skills4genius programme (Santos et al., 2017) or the Creative Soccer Platform (Rasmussen and Østergaard, 2016) – “show some promise and suggest creativity is trainable” (De Sa Fardilha and Allen, 2019, p. 20). However, De Sa Fardilha and Allen (2019) point out that the definitions and assessments of creativity in the field are heterogeneous and pronouncedly emphasize cognitive aspects. Consequently, it still remains unclear exactly which measurable aspects of “creativity” presented in the current literature can be trained and, more generally, whether respective studies approach the same aspect or completely different entities subsumed under the same label of “creativity.”

The objective of the present review is thus twofold. First, it aims to provide an overview of the literature addressing creativity in team sports with the main focus—differing from and complementing the recent review by De Sa Fardilha and Allen (2019)—to characterize studies in terms of their underlying conceptualization and operationalization of creativity. Evidently, in certain cases, assigning studies to theoretical approaches might be challenging as some studies may be based on a mixture of approaches or may focus on an empirical contribution without a clearly defined theoretical link. In these cases, the classification apparently requires to read between the lines in order to come up with a reasonable assignment. However, rather than clear-cut statements that a specific study A belongs to the theoretical approach B, our aim is provide a comprehensive view of the entire field of research focussing on the question which approaches are pursued per se and which contributions have been made from which perspective. The derived aggregation is thought to offer a valuable framework for future discussions on the rather elusive notion of creativity in team sports. Moreover, disentangling the relevant distinctions between pursued approaches may facilitate the derivation of well-grounded recommendations for sport practice from the current scientific literature on the topic. Following the Teaching Games for Understanding classification (Butler et al., 2003), only studies on invasion games will be considered (e.g., football, basketball, ice hockey). No further restrictions in regards to population, study design or outcome variable are applied. Second, the conceptualization of creativity as a player's DT ability will be critically discussed on the basis of a comparison with existing summaries of empirical findings (e.g., Memmert, 2011a, 2013, 2014, 2015a,b, 2017; Memmert and König, 2019; Memmert and Roca, 2019). In line with a relational concept of creativity and a functional approach to behavioral control, an alternative point of view on the issue of developing creative players in team sports will be proposed.

## Method

**Figure 1:**
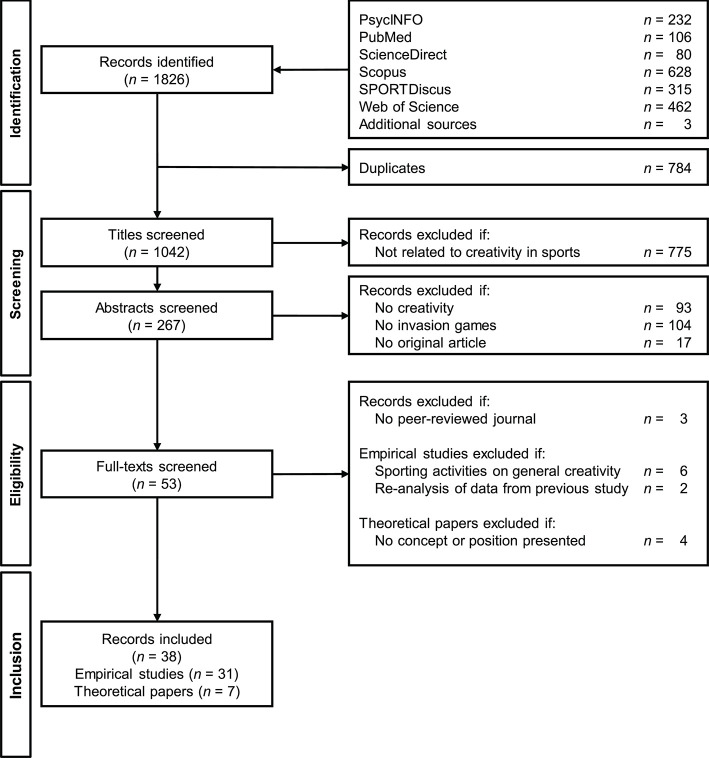
PRISMA flow diagram for the literature search.

The present systematic review was conducted in accordance with the *Preferred Reporting Items for Systematic Reviews and Meta-Analyses* guidelines (PRISMA; Moher et al., 2009), following the steps of identification, screening, eligibility inspection and inclusion of relevant studies as illustrated in Figure 1. For in-detail information on the implementation of the reporting guideline items, see PRISMA checklist in Appendix A.

### Inclusion and Exclusion Criteria

Studies were included if they (a) referred to creativity in team sports, specifically in invasion games (Butler et al., 2003), (b) were original studies published in peer-reviewed journals, and (c) were written in English language. Due to the focus on invasion games, studies referring to creativity in other sports and movement contexts, for instance in individual sports or dance, were excluded. Furthermore, studies on the influence of sporting activities on general creativity (e.g., Bowers et al., 2014) were not included. Both empirical and theoretical papers were examined. However, theoretical papers were only included if they presented a concept, methodological approach or differentiated position directly related to creativity in team sports.

### Identification

For the literature search, six academic databases were used: PsycINFO, PubMed, ScienceDirect, Scopus, SPORTDiscus, and Web of Science. The last search was conducted on June 17th, 2019. The following keyword logic was applied: [creativ^*^ OR “divergent thinking”] AND [sport^*^ OR soccer OR football]. If available, filter options restricting the search to peer-reviewed, academic journal articles published in English were applied (for full electronic search strategy, see Appendix B). In total, 1,823 records were identified through database search, and subsequently exported to EndNote. Additionally, three articles meeting the inclusion criteria were found from backward and forward citation searching; that is, checking the reference lists of included full texts and inspecting articles citing them. Two of these articles were available on the web and one was kindly shared by the author.

### Screening

After removing duplicates, the remaining 1,042 studies were screened on title level. At this stage, articles were excluded if the title clearly revealed a focus irrelevant to the questions addressed in this review (e.g., creative fashion in sports). Due to the frequent use of the term “creative” in a wide range of contexts, this step seemed appropriate, and led to the exclusion of 775 articles. The remaining 267 articles were subsequently examined on abstract level. In this step, articles were omitted from the review if the study did not focus on creativity (i.e., the term creativ^*^ was used without direct relevance to the study), the study did not refer to invasion games, or the paper was identified as a review paper rather than an original article.

### Eligibility

In the eligibility phase, the remaining 53 articles were screened at full-text level. In a first step, three articles were excluded because they were not published in a peer-reviewed journal. The 50 articles retained consisted of 39 empirical studies and 11 theoretical papers. In order to meet the inclusion criteria mentioned above, empirical studies were excluded if the effect of sporting activities on general creativity was investigated (*n* = 6) or data from a previous study were re-analyzed (*n* = 2). Furthermore, theoretical papers were only included if they presented a differentiated position. Consequently, four further articles in which the importance of creativity in team sports was more generally discussed were excluded (namely: Duriček, 1992; Bjurwill, 1993; Aggerholm et al., 2011; Machado et al., 2019).

### Inclusion

Ultimately, 38 articles were included in the present review, consisting of 31 empirical studies and seven theoretical papers. In order to comprehensively categorize these records in terms of their conceptualizations and operationalizations of creativity, three comparative elements on different levels were considered. First, the four Ps scheme (Rhodes, 1961) was applied; classifying the studies as mainly emphasizing person-, process-, product- or press-related aspects of creativity. Second, the context in which creativity is manifested was identified; either relating to game situations or to a wider context beyond game-performance as a developmental resource. Third, the task used to measure creativity was identified; for instance, the application of a video-based task or game-test situations on the field to assess DT ability. Additionally, information relating to the author(s), year of publication, study type, participants and investigated sport were extracted. Finally, the main findings and, if explicitly stated, recommendations for sports practice were briefly summarized.

## Results

Table 1 presents the included studies, their characterizations and their summaries. Studies were first classified by their four Ps' focus, with only person, product and process represented in the reviewed literature. Additionally considering the manifestation context as a comparative element, the studies could be grouped into nine categories that are reasonably distinguishable in terms of their conceptualization, or recognized definition, of creativity. Within each of these categories, studies were further classified in respect to the creativity tasks examined and then ordered chronologically by the publication year. Furthermore, the investigated sport, the study type, main findings and, if available, recommendations for sports practice are reported. Two studies that do not fall into these categorizations, focusing on coaches' conceptualizations of creativity, are not included in the Table 1.

**Table 1:** Studies on creativity in invasion games classified by their conceptualization of creativity, ordered by four Ps' focus (person, process, product, press), conceptualization, creativity task, and publication year (alphabetically for studies published in the same year).

**Four Ps' focus**	**Conceptualization**	**Creativity task**	**Author(s)**	**Sport**	**Study type**	**Main finding**	**Recommendations for sports practice**
Person	Creativity as a domain-specific DT ability	DT in game-test situations (field -based)	Memmert (2006)	Invasion games (diversified: playing with foot, hand, and hockey stick)	Quasi-experimental (field-based)	1. Diversified sport enrichment programme enhanced team ball sport-related DT more in gifted (vs. non-gifted) children.2. Differences in attention (inattentional blindness task) are discussed as explanation.	
			Memmert (2007)	Invasion games (diversified: playing with foot and hand)	Quasi-experimental (field-based)	Six-month attention-broadening training programme enhanced children's DT, which suggests the trainability of creativity in team sports.	To encourage deliberate play and experiences across different sports games and to avoid restrictive tactical instructions.
			Memmert and Roth (2007)	Football, handball, and field hockey	Quasi-experimental (field-based)	1. Both non-sport-specific and sport-specific training approaches improved children's DT.2. Transfer of DT improvements across ball games was observed.	To favor non-sport-specific concepts in teaching invasion games.
			Greco et al. (2010)	Basketball	Quasi-experimental (field-based)	Deliberate play programme enhanced both DT and game intelligence measures.	To encourage involvement in unstructured, play-oriented situations.
			Memmert (2010)	Football	Validation and dynamic performance diagnostic	Game-test situations can be considered as an objective and valid tool to assess tactical creativity (operationalised as DT) in talented youth football players.	To use game-test situations as a tool to compare tactical creativity of players across talent bases.
		DT in video-based tasks	Memmert (2011b)	Handball	Cross-sectional (skill level, attention and age)	1. Attentional processes (less inattentional blindness) are related to higher DT-scores.2. Development in DT from age 7–10 to 10–13 does not increase linearly, as a stagnation between 10 and 13 was observed.3. A moderate correlation and similar paths of development between general and sport-specific DT were found.	To promote a wide breadth of attention at an early age, especially in beginners' trainings, and to refrain from giving advice during games.
			Memmert et al. (2013)	Football	Experimental	Induced motivational orientation toward promotion (vs. prevention) leads to higher DT scores.	
			Furley and Memmert (2015)	Football	Correlational	No correlation was found between domain-general working memory capacity and football-specific DT.	
			Fink et al. (2018)	Football	Experimental (EEG)	Mentally generating football actions was generally associated with decreases in EEG alpha power at parietal and occipital sites. When instructed to imagine original (vs. conventional) actions, decreases were less pronounced.	
			Furley and Memmert (2018)	Football	Experimental	Priming amateur football players with famous creative football stars enhanced their football-specific DT.	To use videos of creative players prior to training/ match.
			Hüttermann et al. (2018)	Football	Experimental	Inducing a situational promotion focus and negative performance expectations by task instruction enhanced football-specific DT.	To foster creative behavior with promotion-oriented instructions.
			Roca et al. (2018)	Football	Correlational	Individual differences in football-specific DT were underpinned by differences in visual search strategy with more fixations of shorter duration and toward more informative locations.	To design practice environments that promote a wide breadth of attention.
			Fink et al. (2019)	Football	Experimental (fMRI)	Generating conventional (vs. original) actions was related to stronger activations of the left-lateralized networks. Higher originality scores were associated with smaller activation differences between conditions (conventional vs. original).	
			Hüttermann et al. (2019)	Football	Correlational	Individual differences in attentional capability (attention-window-task) and expertise level explained a significant amount of variance in participants' DT.	To develop attention-training programmes and consider attentional capability as a potential selection criterion.
		Handball-specific inattentional blindness task	Memmert and Furley (2007)	Handball	Experimental	Tactical “if-then-rules” led to a narrower breadth of attention resulting in not noticing obviously unmarked players with potentially negative effects on creativity.	To promote a wide focus of attention, especially in beginners' trainings.
			Memmert et al. (2010)	Basketball, football, handball, and field hockey	Survey (retrospective)	Highly creative offensive (vs. less creative defensive) professional players spent more time in both unstructured play in their main sport and sport-specific training through their career. No differences in the number of other experienced sports were found.	To encourage both playful activities to enhance motivation and creativity and the specific practice necessary to adapt to task-relevant demands.
		Coach ratings of players' creative ability	Hendry et al. (2018)	Football	Survey (prospective)	No correlations were observed between the amount of deliberate football play and players' creative skill ratings at the age of 15 (academy), 17 (young professional), 20 (adult professional).	To favor sport-specific practice to unstructured play in order to develop skill (incl. creative skills).
	Creativity as an everyday-relevant quality	General creativity-tests (Torrance, 1966; Urban and Jellen, 1995)	Kováč (1996)	Football	Cross-sectional	Scores in general creativity tests were moderately correlated with football performance.	To foster creativity in sport-talent development schools.
			Kováč (1998)	Football	Quasi-experimental	A psychological creativity training had a small, but positive effect on creativity-test scores.	
		rCAB (incl. DT-test adapted to sport-related problems)	Richard et al. (2017)	17 sports (incl. football, hockey, rugby, ultimate frisbee, and water polo)	Survey (retrospective)	Expert (vs. intermediate vs. advanced) athletes exhibited a higher level of creativity, especially in DT. Engagement in different sports at the recreational level was related to higher levels of creativity.	To encourage sampling many sport activities and promote creativity as a life skill.
	Creativity as a higher-order disposition		Santos et al. (2016)		Theoretical paper	The Creativity Developmental Framework is presented as a holistic model that integrates different concepts (e.g., non-linear pedagogy, differential learning) with the goal of fostering players' creativity in five incremental stages.	To emphasize intrinsically motivating diversified play in early stages, followed by a gradual specialization in later stages.
		CBATS in small-sided games (3 vs. 3)	Santos et al. (2017)	Football	Experimental (field-based)	1. The Skills4Genius programme enhanced both in-game creative components of the CBATS and general DT of children.2. A strong correlation between general DT and in-game creative behavior was found.	To use the Skills4Genius programme to foster children's creative thinking in everyday life and creative behavior in the game.
		CBATS in small-sided games (5 vs. 5)	Coutinho et al. (2018)	Football	Quasi-experimental (field-based)	A differential-learning programme enhanced in-game creative components of the CBATS for youth football attackers.	To use differential learning in order to improve in-game creative behavior.
			Santos et al. (2018)	Football	Experimental (field-based)	Differential learning applied to small-sided games enhanced in-game creative components of the CBATS.	To use differential learning in small-sided games for training and physical education.
	Creativity as an embodied potential		Hopsicker (2011)		Theoretical paper	In a developmental pathway of highly creative athletes, deliberate practice is deemed crucial in the preparation phase, followed by building a risk-taking attitude.	To invest in deliberate practice in order to develop a broad range of physical skills.
			Campos (2014)		Theoretical paper	In-the-moment creativity is described as the potential to respond to physical challenges in spontaneous and imaginative ways that is founded in carefully cultivated skills.	
			Martin and Cox (2016)	Basketball	Qualitative (biographical single-case)	Childhood experiences, such as playing a variety of sports and an extreme amount of self-initiated practice, led to creative on-court expertise. Anticipating future game demands, the athlete's skill repertoire was diversified even though these skills were not necessary early on.	
Process	Creativity as a general cognitive component		Vestberg et al. (2012)	Football	Correlational	Study 1: Higher (vs. norm group vs. lower) division football players scored higher on DF.Study 2: A correlation between DF-test scores and the number of goals and assists 2 years later was found.	To consider standardized neuropsychological tests as a potential talent-selection instrument.
		Design Fluency (from D-KEFS test battery)	Lundgren et al. (2016)	Ice hockey	Correlational	Ice hockey players (vs. standardized norm group) scored higher on DF. No differences between elite and lower division players were found.	To consider game-relevant cognitive functions for talent identification in ice hockey.
	Creativity as an exploration and production of novel and functionally efficient behaviors		Hristovski et al. (2011, 2012)		Theoretical paper	Creative behavior is conceptualized as the process of exploration and discovery of novel functional movement patterns, relative to one's own action landscape or the socio-cultural landscape, with a focus on manipulating task constraints in order to enhance exploratory behavior.	To design practical tasks that enhance exploratory behavior by relaxing key constraints or suppressing habitual actions.
		Exploratory behavior in small-sided games	Torrents et al. (2016)	Football	Experimental (field-based)	When manipulating number of players (4 vs. 7/4 vs. 5/4 vs. 3), a numerical disadvantage led to more exploratory behavior.	To enhance exploratory behavior by introducing constraints that suppress actions in one's comfort zone.
	Creativity as a developmental resource	Focus group interview with players and semi-structured interview with the coach	Rasmussen and Østergaard (2016)	Football	Qualitative	A creativity-stimulating environment in organized youth football was established with the Creative Soccer Platform. Players had the opportunity to experience and discover new actions without fear of making mistakes.	To organize trainings that allow judgement-free exploration of unusual action possibilities.
			Rasmussen et al. (2019b)		Theoretical paper	Creativity is conceptualized as a developmental resource in training activities. Rather than as a trait, creativity is understood as a dynamic quality of action located in the transaction between the player and the situation.	To stimulate the experience of exploring unusual action possibilities in safe, playful and autonomy-supportive environments.
Product	Creativity as original and functional motor actions		Orth et al. (2017)		Theoretical paper	Challenging traditional accounts of creativity focusing on the (enaction-independent) generation of ideas, an alternative viewpoint is presented that conceives creative actions as a product of individual, task and environmental constraints emerging in the act.	To promote exploration by manipulating constraints in order to increase movement variability and, thus, the probability of finding creative solutions.
	Creativity as a feature of actions (enabled by DT)	Expert rating of actions on a creativity scale	Kempe and Memmert (2018)	Football	Match analysis	1. Actions of more (vs. less) successful teams were rated as more creative.2. More (vs. less) successful teams scored more highly creative goals.	To specifically train creativity in professional football.

*CBATS, Creative Behavior Assessment in Team Sports; DF, Design Fluency; D-KEFS, Delis–Kaplan Executive Function System; DT, Divergent Thinking; rCAB, Runco Creative Assessment Battery; TTCT, Torrance Tests of Creative Thinking*.

After providing the reader with a descriptive overview, the following sections aim to grasp the relevant distinctions between the identified conceptualizations of creativity in team sports. In the narrative synthesis, the most common approach—creativity as a person's domain-specific DT ability—will be taken as a starting point. From there, comparisons will be drawn within the person-related conceptualizations of the four Ps, before discussing process- and product-related approaches.

### Descriptive Overview

Overall, some broad patterns can be identified in the reviewed literature. In terms of the four Ps, most studies captured creativity as a quality of the person; more specifically, as an ability “possessed” by the player (29 of the 38 studies reviewed). Regarding authorship, Memmert and colleagues have contributed a substantial number of studies to the topic of interest (19 of the 38 studies reviewed; 17 of the 31 empirical studies). Notably, creativity was mainly conceptualized as a domain-specific DT ability (17/38). Alternative conceptualizations presented in more than two articles include creativity as: a higher-order disposition (4/38), an everyday-relevant quality including cognitive and personality aspects (3/38), an embodied potential (3/38) and an exploration and production of novel functionally efficient behaviors (3/38).

### Creativity as a Property of the Person

#### Creativity as a Domain-Specific DT Ability

In the most widespread approach to creativity in team sports (17/38), “having tactical creativity” (Memmert et al., 2010, p. 3) is described as an ability of an individual player. This concept is usually introduced by translating convergent and divergent thinking to the terms of “game intelligence” and “tactical creativity”, respectively, as they are frequently used in sports practice. In this regard, game intelligence is defined as the ability to find the best solution to a given problem, whereas creativity is understood as the more or less developed ability to generate a variety of options—described as “surprising, original and flexible” —in response to a game situation (Memmert, 2010, p. 199). Thus, beyond its measurement through DT tasks, creativity is explicitly equated to DT on a conceptual level. Moreover, an individual's DT ability is presumed to manifest itself in creative actions “across different situational contexts” within team ball sports (Roca et al., 2018, p. 1, 2). In this sense, tactical creativity is proposed to be a moderately domain-specific ability, with predicted transfer effects across different invasion games (Memmert and Roth, 2007). Notably, such an understanding explicitly decouples creativity from motor skill: “Unlike motor competencies, it is possible to train tactical creativity independently of the movement techniques” (Memmert and Roth, 2007, p. 1429). Conceptually, this approach is inspired by Sternberg and Lubart's (1992) integrative model of creativity (cf. Memmert, 2015b). According to this model, team sports represent a domain for creative abilities that, as described by Fink et al. (2018), is considered to be a “worthwhile field” (p. 118) to study creativity in an ecologically valid way.

Empirically, the importance of promoting a wide breadth of attention, referring to “the number and range of stimuli that a person is able to attend to at any one moment in time … [including] stimuli that initially appear to be irrelevant” (Memmert, 2011b, p. 94, 95), has been repeatedly highlighted as a means to enhancing DT in team sports (Memmert, 2006, 2007, 2011b; Memmert and Furley, 2007; Hüttermann et al., 2019). With a focus on attentional processes, Memmert and Furley (2007) argue that “the more elements a person can focus on simultaneously, the more likely he is to make a greater variety of tactical decisions” (p. 367). Moreover, creativity is conceived as a competency “that cannot much be improved upon in later training phases” (Memmert and Roth, 2007, p. 1423); an aspect that has been investigated empirically (Memmert, 2011b) and that suggests the importance of training at an early stage.

In the reviewed studies, two distinct evaluation instruments were used to measure tactical creativity as a DT ability: game-test situations and video-based tasks. Both evaluation instruments result in a DT score for each individual player. Table 1 reveals that game-test situations were used in early studies on the topic (2006–2010) and mainly to assess the creativity of young children (cf. Memmert, 2006, 2007; Memmert and Roth, 2007), whereas video-based tasks have been pre-dominant in the recent years (2011–2019). Game-test situations are a field-based assessment. Players repeatedly perform basic game tasks, with a standardized number of players and rules. The tasks emphasize basic tactical problems that occur across various invasion games (e.g., “identification of gaps”, for in-detail descriptions of respective tasks, see Memmert, 2006, 2007, 2010; Memmert and Roth, 2007). Behaviors in the tasks are videotaped and subsequently rated by experts. Each player is evaluated on a DT scale that regards two factors: originality (the “unusualness of the children's ideas”, Memmert and Roth, 2007, p. 1426) and flexibility (the diversity of solutions). It is emphasized that “the evaluation of technical skills must be avoided” (Memmert, 2015b, p. 82) and that the evaluation should only refer to the intended solutions. Furthermore, the expert rating is described as “concept-oriented”, meaning that the experts had been trained beforehand to use specific criteria to assess original and flexible behaviors: “Only experts showing a high reliability as measured against a “golden standard” of ball games experts were chosen” (Memmert and Roth, 2007, p. 1426). Each player is usually rated by three experts independently and then these ratings are averaged to form a creativity score. In studies examining different game tasks (e.g., “identification of gaps” and “orienting and supporting”) and/or different sports (e.g., football, handball and field hockey), all scores are generally averaged together for an overall creativity value (Memmert and Roth, 2007).

In sport-specific video-based DT-tests, participants are typically shown 20 video clips of attacking game situations that are temporally occluded at key moments, usually by freezing the final video frame. The participants' task is to imagine themselves as the player with the ball and to name (or write down) as many options as they can think of within a given time frame (e.g., in 45 s; Memmert et al., 2013; Furley and Memmert, 2015, 2018; Hüttermann et al., 2018, 2019). Here, all three DT components of fluency, flexibility and originality are regularly scored for creativity measures. While indeterminable in game-test situations (cf. Memmert and Roth, 2007), fluency can be evaluated in video-based tasks as the number of options generated for each scene. For the flexibility score, each response is grouped into a class (e.g., shot on goal, cross, short pass, etc.) and one point is given for every distinct class in which a player has generated a solution. For originality, each proposed solution is rated by experts on a Likert scale (1–5) and then all ratings are averaged for an originality score. In the standard procedure used in the reviewed studies, the three components (fluency, flexibility and originality) are first analyzed separately and subsequently averaged after z-transformation to obtain the player's overall DT score (Memmert, 2011b; Memmert et al., 2013; Furley and Memmert, 2015, 2018; Hüttermann et al., 2018, 2019; Roca et al., 2018).

#### Creativity as an Everyday-Relevant Quality

Similarly framing creativity as an important attribute of players and relying on DT, the position of Kováč (1996, 1998) and Richard et al. (2017) depicts creativity as a value demonstrated beyond the pitch. Here, DT is not related to game situations but rather captured in a wider context as a “developmental resource” (as termed by Rasmussen et al., 2019b, p. 491). This perspective becomes clear when looking at the creativity assessment used by Richard et al. (2017) in which, from a battery of creativity assessments (Runco Creative Assessment Battery: DT, Creative Attitude and Values, Creative personality, Creative Activity and Accomplishment Checklist), a DT-test was adapted to sports problems. An example item is as follows:

Your coach announces an extra practice tomorrow because he is not satisfied with your performance today. Unfortunately, you have a big school project due the day after, and that will require a full day to complete. You can't miss the extra practice but you need the day for your project. What are you going to do? Think of as many ideas as you can! (p. 68).

The test scoring is similar to that used in Memmert's DT-tests (e.g., Memmert et al., 2013) as described above: fluency, flexibility, and originality of generated ideas.

#### Creativity as a Higher-Order Disposition

In the holistic creativity developmental framework proposed by Santos et al. (2016) and respective empirical studies (Santos et al., 2017, 2018; Coutinho et al., 2018), creativity is defined as a higher-order disposition, valuable to players in game situations and beyond; thus, less restricted to a specific domain. In this vein, pedagogical aspects of developing creative potential are additionally emphasized (Santos et al., 2017).

This latter focus is well reflected in the creativity assessment utilized in the respective studies with this viewpoint (Creative Behavior Assessment in Team Sports; CBATS). In such evaluations, the behavior of players in small-sided games is video recorded and the actions (pass, dribbling, shots) are classified according to “creative components” (Santos et al., 2018, p. 15). These creative components are terminologically inspired by DT concepts, yet interpreted in a slightly different way than by Memmert and colleagues (e.g., Memmert et al., 2013). Namely, the following four components are distinguished: fluency (ability to execute as many effective movement actions as possible), versatility (ability to produce non-standard actions), originality (ability to generate new and unique actions), and attempts (any effort to perform different actions, even non-effective movements). The components are assessed by first dividing the notated actions into groups of successful and unsuccessful as well as standard and non-standard. Subsequently, the components are quantified as follows: fluency as the count of successful, but standard, actions; versatility as the count of non-standard actions; originality as the count of actions that players performed only once; and attempts as the count of non-successful actions when trying a non-standard action. On this basis, it seems clear that the CBATS is not an instrument for evaluating performance in its narrow sense, but more so a quantitative characterization of game behavior that recognizes the pedagogical value of trying out different actions when playing games. Empirically, Santos et al. (2017) reported a positive correlation between creative behavior in small-sided games and a creative thinking test (TTCT, Figural Version).

#### Creativity as an Embodied Potential

Creativity as a capacity in team sports has also been described based on phenomenological accounts of sporting experience (Hopsicker, 2011; Campos, 2014). This understanding refers to the same manifestation context as Memmert (e.g., Memmert, 2010)—namely, in game situations—however from a fairly different perspective. Campos (2014) offers a rich phenomenological description of “effective creativity under constraint” (p. 54); that is, creativity within the rules that is directed toward the goal of the game, expressed through his or her body and delimited by one's skills. Creativity is described to be enacted by the player in the course of a game situation and is hence termed “in-the-moment creativity” (p. 55). The ability to respond to challenges encountered during a game in spontaneous and imaginative ways is suggested to rely on both specific movement skills and imaginative potential. It is emphasized that this understanding does not imply that the mind “imagines”, and the body “obeys.” Rather, the mastery of skills liberates imagination, which is defined as the “capacity to conceive possible courses of action that must not only be visualized but must be felt to be possible” (p. 67). Similarly, for creativity in sports, Hopsicker (2011) highlights the importance of building a “broad array of physical skills” (p. 116) that give the athlete “more potential choices of action—a more diverse menu that allows him to perform his skills in new and creative ways” (p. 123). According to Campos (2014), the athlete is thus understood as the “bodymind that moves and thinks in a continuous act” (p. 56). Despite similarly recognizing creativity with the “imaginative conception” (p. 66) of alternative solutions, this framework seems to be fundamentally incompatible with the previously described approach of capturing creative ability; particularly in the static fashion of a DT video task that neglects embodied actions (Memmert et al., 2013).

As the three studies that conceptualize creativity as an embodied potential exclusively focus on theoretical considerations, no methods for quantifying creativity can be reported.

#### Creativity as a General Cognitive Component

When ordering the included studies with respect to the conceptualization of creativity as a more or less cognitive ability, on the opposite end of the spectrum to the embodiment approach sketched above, creativity is also seen as a purely cognitive feature that is completely decoupled from sport-specific contexts (Vestberg et al., 2012; Lundgren et al., 2016). In comparison to the approaches presented so far, here, the construct of creativity does not stand alone. Rather, it is subsumed as an element of the executive functions: “assessing players' general executive functions including on-line multi-processing such as creativity, response inhibition, and cognitive flexibility” (Vestberg et al., 2012, p. 1). The respective articles have been included for the review since Kempe and Memmert (2018) cited Vestberg et al. (2012) for showing that “creativity is a predictor of individual success” (p. 2422) in football. More precisely, Vestberg et al. (2012) report a significant partial correlation between test scores in a standardized neuropsychological assessment and the number of goals and assists two seasons later. In this study, the Design Fluency test from the Delis-Kaplan Executive Function System was used, which is described to cover a “creativity/planning aspect” (p. 2). As a paper-pencil test, the task is to combine dots in as many different combinations as possible under time pressure (i.e., within 60 s). According to the authors, “on-line multi-processing” (p. 1) and “fast creativity” (p. 3) are considered to be important cognitive components for team sports. While this neuropsychological test is clearly domain-unspecific, certain parallels can nevertheless be recognized with the DT-test of Memmert et al. (2013); specifically, the component of fluency in the conceptualization of creativity.

## Creativity as a Process

### Creativity as an Exploration and Production of Novel and Functionally Efficient Behaviors

From the perspective of ecological dynamics, Hristovski et al. (2011, 2012) present a model of creativity that focusses on the exploratory process and discovery of novel (or atypical) performance solutions. The novelty of the solution or movement pattern is understood in two reference frames: novel relative to either the performer's intrinsic dynamic action landscape or the socio-cultural action landscape of the domain. Therefore, as opposed to framing creativity as an individual's characteristic (i.e., a personal ability or trait), the matter of interest is rather how creative (i.e., novel and functional) solutions emerge as a consequence of task constraints.

In this perspective, the exploration of different action configurations—within the multitude of states available in the performer-environment system—is a prerequisite for discovering novel movement patterns. The exploratory process is described as “subsequent realization of a large number of movement configurations” (Hristovski et al., 2011, p. 187) or, metaphorically, as a “hopping between attractors on different hierarchical levels” (Hristovski et al., 2012, p. 31). These hierarchical levels refer to the exploratory breadth, implying that the system can explore different configurations within one mode of action or encompass different classes of action. As constraints acting on the performer-environment system shape exploratory behavior, the focus is put on how to manipulate task constraints in order to enhance the exploratory breadth, and thus increase the probability of discovering novel performance solutions, or “action insight[s]” (Hristovski et al., 2011, p. 195). In regards to practice, two strategies based on “relaxing task constraints” (Hristovski et al., 2011, p. 175) have been proposed: The first strategy, termed direct relaxation, regards changing task constraints such that “the number [of] affordances that can satisfy goal constraints increases” (p. 175); in the second, termed indirect relaxation, habitual actions are suppressed by task constraints forcing a larger exploration and “new affordances to emerge” (p. 175). Furthermore, in respect to discovering innovative performance-solutions, Hristovski et al. (2011) discuss the emergence of solutions that are both novel relative to a specific socio-cultural context and highly efficient in reaching the task goal in a “cross-fertilization” process (p. 194); that is, by blending movement patterns form different domains or disciplines and thus re-inventing a task-solution in a novel context.

Translating this approach to empirical research in team sports, Torrents et al. (2016) investigated the effect of changing the number of teammates and opponents (as key task constraints) on the exploratory behavior in small-sided games. Here, the question of practical relevance was not how to directly develop creativity of a player, but how to “facilitate the emergence more of varied behavior” (p. 4) and “promote a search in different parts of the problem space” (p. 11). While observations of small-sided games were similarly used in the studies of Santos et al. (e.g., Santos et al., 2018) sketched above in the context of creativity as a person's higher-order disposition, the methodological approaches clearly differ. In the study conducted by the Torrents group, in order to quantify exploratory behavior under different task constraints, actions were defined on a scale of 51 categories for every 1 s time interval (Torrents et al., 2016). This resulted in a 51-component binary vector representing action configurations for every time window. Principal component analysis was performed to identify different types of action configurations, and average dynamic overlap q_d_(t) was taken as a measure of exploratory behavior, capturing the similarity of configurations with increasing time lags. The stationary value of dynamic overlap q_stat_ was then compared across experimental conditions (i.e., number of players). For example, if players repeated the same action over the observation time, the value would be close to 1; whereas, if players explore all possible combinations of actions, the value would be close to 0. In terms of practical recommendations, the authors suggest that numerical inferiority in small-sided games—more generally, inducing discomfort by suppressing habitual actions—promotes the exploration of a variety of actions and consequently can be expected to lead to the discovery of creative, meaning novel and functionally efficient, behaviors.

### Creativity as a Developmental Resource

In a sport-pedagogical intervention study (Rasmussen and Østergaard, 2016) and a theoretical paper (Rasmussen et al., 2019b), Rasmussen et al. put forward a conceptualization of creativity that encompasses a broader context. It emphasizes the “developmental and experimental benefits of creative activities” (p. 491)—that is, of experimenting with unusual action possibilities—for all players, at all levels, in and beyond game performance. Creativity is described as a “developmental resource in training activities” (p. 492), playing an important role in learning, enjoyment and breaking with limiting routines. Theoretically, creativity is not attributed to a person in the form of an ability or a trait, but is rather seen as a “dynamic quality of action” (Rasmussen et al., 2019b, p. 491) located in the transaction between the person and the environment. In this regard, emphasizing moment-to-moment player-environment interactions in the explorative process, conceptual overlaps with the contributions of Hristovski et al. (2011, 2012) can be identified. However, the position of Rasmussen et al. clearly contrasts the former by extending the focus beyond the search for novel in-game performance solutions. Instead, it is the playful process of exploring unusual action potentials— “as a means rather than an end” (p. 497)—that is deemed creative.

This understanding is well-reflected in the study of Rasmussen and Østergaard (2016). A creativity stimulating training was implemented by establishing a judgement-free environment that allowed players to explore unusual action possibilities, which would normally be avoided due to conventional norms. After the intervention, no formal creativity assessments were conducted; rather, the players' experiences were recorded qualitatively and an interview was conducted with the coach.

## Creativity of the Product

### Creativity as Original and Functional Motor Actions

Theoretically related to the above described perspective of creativity as a process, specifically as the exploration and production of novel and functionally efficient behaviors, Orth et al. (2017) similarly present a point of view on creative actions rooted in dynamical systems and ecological approaches. The theoretical paper emphatically criticizes cognitive accounts of creativity. More specifically, the underlying assumption that creative ideas are first generated by an individual in his or her mind and subsequently enacted in observable behaviors is challenged. Based on this opposing perspective, measuring creativity with a video-based DT-task (cf. Memmert et al., 2013) that explicitly targets the generation of ideas in response to a stimulus seems fundamentally irrelevant. It is rather argued that creative solutions “emerge *in* the act rather than *before* … [and] are as much a product of individual constraints as they are of the task and environment constraints” (p. 1). Consequently, the term creative is used “as a descriptive for unfolding actions that are original (relative to the individual or group) and functional (i.e., they support task success)” (p. 2). Creative actions are thus conceived as adaptive motor solutions that are exceptional in originality relative to alternative solutions (i.e., statistical rareness). Such actions are not expected to be found when explicitly looking for creative solutions; rather, it is predicted that creative actions emerge from the movement variability that arises when aiming to satisfy changing constraints.

Based on this conceptual shift, Orth et al. (2017) present an operational framework to experimentally study the emergence of creative actions when searching for functional solutions under constraints. It is proposed to use “motor tasks that invite participants to actively search solutions to a motor problem across a series of attempts” (p. 5). With this methodological strategy, participants' behavioral repertoire (i.e., their stable task-solutions), as assessed in a scanning procedure, before and after a practice intervention can be compared. “Identifying new solutions that meet a criterion for task success (functionality) and have statistical level of rarity for the particular workspace (originality) is a straightforward and theoretically consistent methodology for studying motor creativity” (p. 5).

### Creativity as a Feature of Actions (Enabled by DT)

The understanding of creativity as an emerging product, as sketched above, seems to be completely incompatible with its conceptualization as a person's ability—as it has been reported to be mainly approached by Memmert and colleagues (e.g., Memmert et al., 2010)—especially when focusing on a person's cognitive ability in terms of DT. However, it should be noted that there is one study by Memmert et al. that considerably differs from those presented within the domain-specific DT ability category: namely, the study by Kempe and Memmert (2018). In this paper, instead of assessing individual abilities, creativity is approached as a property of actions. Here, experts rated the last eight actions before a goal in open play in three major international football tournaments on a creativity scale (0–10). Goals with at least one action rated 8+ were defined as “highly creative” (p. 2421). The analysis revealed that the actions of more successful teams were rated as more creative and that the more successful teams scored more highly creative goals. These findings are interpreted by the authors as “empirical evidence that creativity [in terms of creative actions] is a decisive factor for success in soccer” (p. 2422).

However, in stark shift, Kempe and Memmert (2018) switch back to an ability perspective by emphasizing that “to enable players to perform those creative actions, several studies on how to best train creativity in soccer had been conducted in recent years … [and that] results could demonstrate that creativity can be learned as well as trained” (p. 2419), referring to the presently reviewed studies in the respective section (i.e., Kováč, 1998; Rasmussen and Østergaard, 2016; Santos et al., 2016, 2018). Consequently, as for sports practice, the authors recommend to train “creativity” (defined as DT, p. 2419) through implementing training principles proposed by Memmert (2015b; Tactical Creativity Approach) and—linking to the findings of Vestberg et al. (2012)—to consider additional cognitive training interventions. Although based on an analysis of game actions in terms of creative products, by ultimately contextualizing their study in line with research capturing creativity as a DT ability, Kempe and Memmert (2018) conclude that their study “provides an empirical basis for the ongoing debate on the importance of creativity training in football” (p. 2419).

## Coaches' Conceptualizations

In addition to the conceptualizations presented so far, in two studies, the focus was laid on the exploration of coaches' views of creativity. Leso et al. (2017) used a questionnaire (closed response items) to investigate coaches' associations of creativity and game intelligence with different attributes. In this study, coaches seem to relate creativity to a kind of “magical thinking” (p. 182). Aiming to grasp an in depth understanding of practitioners' nuanced views on creativity, Rasmussen et al. (2019a) conducted an expanded qualitative study with a professional football club; results provided 15 metaphors capturing differentiated meanings, benefits and applications of creativity in football. Given the varied meanings of “creativity” that co-exist even within one football club, this finding gives a clear hint of the “difficultly of working with creativity” (p. 13).

## Discussion

Regarding the goal of developing creative players in team sports, the main objective of this review is to facilitate the derivation of well-grounded practical recommendations based on sport-scientific literature. Overall, the review highlights that the conducted studies differ considerably in terms of their underlying conceptualizations of creativity. Aiming to disentangle the pursued lines of thought, nine categories referring to distinct conceptualizations were identified. Relating to the four Ps of creativity research (Rhodes, 1961), most of these conceptualizations can be described as person-related; that is, creativity is understood and assessed as a—more or less developed—quality of the player (e.g., as the player's domain-specific DT ability). Alternative views on the emergence of creative actions were characterized as either process- or product-related.

Beyond the four Ps, conceptualizations were mainly found to differ along two distinct spectrums. The first spectrum regards the notion of creativity as a cognitive ability. Here, a sharp split is apparent between approaches characterizing creativity as a purely cognitive component on one end (e.g., Vestberg et al., 2012) and creativity as an embodied potential on the other end (e.g., Campos, 2014). The second spectrum concerns the context in which creativity is manifested. On one end of this spectrum, studies apply DT to game situations and focus on creativity as an aspect of in-game performance (e.g., Memmert et al., 2010); and on the other end, creativity is seen as an everyday-relevant quality (e.g., Richard et al., 2017). Taken together, this review reveals that, in the field of creativity research in team sports, clearly different entities have been investigated and subsumed under the single label of “creativity.” However, rather than a point of critique, this statement should be mainly understood as a natural consequence of working with the open (Dresler, 2008) and multifaceted (Kaufman et al., 2019; Rasmussen et al., 2019a) construct of creativity. Nevertheless, providing training programmes said to enhance “creativity” in an undifferentiated manner might be misleading.

On a more fine-grained level, in the most widespread approach, creativity is defined and assessed as a player's domain-specific DT ability (e.g., Roca et al., 2018). As exemplified by Kempe and Memmert's (2018) study, this line of thought entails the underlying assumption that it is DT that enables a player to perform creative actions on the field. Initially, Kempe and Memmert (2018) adopt a product-related perspective by asserting that “the level of creativity of the actions” (p. 2419) leading to goals were related to success in international football tournaments, however, later on they interpret creative actions as being enabled by players' individual DT abilities, further highlighting the body of literature “demonstrat[ing] that creativity [as a player's DT ability] can be learned as well as trained” (p. 2419). This body of literature has provided a large amount of empirical evidence supporting that DT—the cognitive capacity to generate many (fluency) different (flexibility) unusual (originality) ideas in response to a game situation—can actually be improved by respective interventions. Specifically, respective studies have revealed effects of a range of factors affecting players' DT. Based on these findings, the Tactical Creativity Approach (Memmert, 2015b) provides a framework for sports practice comprising general methodological principles to foster creativity in team sports by enhancing players' DT ability. Yet, the critical assumption that improving a player's DT ability transfers to creative on-field actions remains untested so far. Besides the lack of empirical support for this assumption, ascribing the potential to perform “creative” actions across situational contexts to a distinct ability of a player—reflected in individual differences in DT and thus focused on the generation of solution ideas explicitly decoupled from one's motor skills (cf. Memmert and Roth, 2007)—can be severely questioned on a conceptual level.

Alternatively, it can be hypothesized that a refined repertoire of sensorimotor skills, which allows for a variety of functional task-solutions to be performed in the course of game situations, increases the probability for actions that—as a consequence—appear more creative to the observer. This alternative point of view is perfectly in line with both a relational concept of creativity as proposed by Westmeyer (1998) and an expert-performance view on creativity (Ericsson, 1999). Specifically, the latter perspective assumes that, with the refinement of task-relevant skills, “adaptation to situational demands will increase and reflect higher levels of creativity” (Ericsson and Lehmann, 2011, p. 488). Rather than being *ascribed* to an overarching cognitive ability, actions are *perceived* as creative relative to the social and cultural context by observers. It should be noted that this relational approach does not hinder the characterization of players who produce such actions as “creative.” However, due to the inherent dependency on the observer's judgement within a specific context, creativity can neither be regarded as a universal feature of an action nor as a property of the player. In the context of team sports, the alternative hypothesis suggests that, unlike *thinking* beyond the expected, *acting* beyond the opponents' expectations is foremost rooted in sensorimotor skill rather than in DT ability. In this view, the competence of a player to actively create solutions in game situations—including behaviors perceived as highly creative—would not be attributed to a pronounced DT ability but rather to his or her rich repertoire of motor skills. To put it differently: As the highly skilled player is less constrained by his/her own skill set, the probability for the emergence of actions that are judged as creative by observers will increase quite naturally.

Beyond Westmeyer's (1998) relational and Ericsson's (1999) expertise-related concepts of creativity, this alternative view also fits with the phenomenological descriptions given by Campos (2014) and Hopsicker (2011), who both emphasize the crucial role of specific movement skills in liberating new possibilities in an ongoing action. Furthermore, it considerably aligns with the rigorous critique on traditional accounts of creativity by Orth et al. (2017), which contest that creative ideas are first generated “in the head” and subsequently executed to solve a problem (see also Withagen and van der Kamp, 2018). Contrary to the DT approach that focusses on an “internal process of generating ideas” (Orth et al., 2017, p. 7) *in response* to a game situation, it would rather be the active and continuous search for promising situations allowing for a greater variety of options that are crucial to be less predictable for the opponent—or in other words, more creative (see also the distinction between creative *thinking about sport* and expressing creativity *in sport* by De Sa Fardilha and Allen, 2019).

Notably, the proposed re-interpretation does not appear to link to a single theoretical perspective on motor behavior; as it aligns with, on the one hand, dynamical-systems (Haken et al., 1985) and ecological approaches (Gibson, 1979) and, on the other hand, computational models of sensorimotor control (e.g., optimal feedback control, Todorov and Jordan, 2002) as well. Based on the prior, an enriched behavioral repertoire could be explained on a scale of functional relationships between the performer and the specific environment (i.e., skill attunement, Araújo and Davids, 2011), resulting in enhanced system degeneracy, “meaning that the individual has developed multiple (and dissimilar) motor solutions for achieving the same outcome or function” (see also Hristovski et al., 2011; Seifert et al., 2016; Orth et al., 2017, p. 5). Based on the latter, within a computational framework, behavioral control can be understood as self-initiated transitions from the perception of a current state to the perception of a desired state (Hossner et al., 2020) with a state transition that fundamentally relies on fine-tuned internal predictions of one's own action effects (i.e., forward models, Franklin and Wolpert, 2011). Moreover, it is important to stress that a relational conception of creativity does not a priori exclude DT as a *potential* resource for a player's creative performance. However, rather than simply equating DT to creativity on a conceptual level and using DT as an undisputable outcome measure, the consequential question of whether an enhanced DT ability transfers to creative actions on the field remains open.

Apparently, the conceptual turn toward a functional-relational foundation of creativity calls for empirical research. In future studies, predictions deduced from both explanations need to be put to empirical test. Namely, if creativity is conceptualized as an ability reflected in individual differences in DT, creative on-field actions, as rated by experts, are predicted to follow improvements in DT. Challenging this assumption, from the proposed functional-relational perspective, it can be predicted that a specific motor skill-related intervention, as compared to a DT intervention, not only leads to actions that are rated as more functional but also as more creative. Such empirical work is currently underway in our research group.

Considering implications for sports practice, the re-conceptualization of creativity as a skill-dependent product reframes the goal of developing creative players: When focussing on solely in-game performance (and not beyond; cf. Rasmussen et al., 2019b), instead of seeking ways to improve players' DT ability (e.g., Memmert, 2015b), the main objective would shift toward increasing *his* or *her* actual options in specific game tasks. In this sense, the issue becomes, in essence, one of complex motor skill learning. When designing practice tasks, this would imply the adoption of a more functional viewpoint; that is, understanding movements as a means to solving tasks in game situations (cf. Hossner et al., 2015). Such an understanding would propose taking task-relevant constraints of specific situations as a starting point and to encourage players to gather specific experiences in regards to task-relevant properties and relations; a notion that perfectly follows a representative learning design (Pinder et al., 2011). More specifically, players should be supported in detecting and stabilizing their own functional—and consequently, potentially novel—task-solutions by systematically manipulating task-relevant constraints in order to “force” players to continuously adapt and explore alternative ways to reach the task goal in a functional manner (for a similar notion, see Hristovski et al., 2011; Orth et al., 2017).

In summary, the present review revealed that a multitude of different aspects have been studied under the subsuming label of “creativity” in sport science. Consequently, in terms of practical recommendations, training programmes that claim to improve players' “creativity” in an undifferentiated manner can be expectedly misleading. In most studies, established concepts of creativity research, such as DT, have been transferred to the domain of sports and measured with well-known creativity tests adapted to sports contexts. On the surface, these approaches seem to cover both the objective of sports practice to find ways to train creativity and the desire of creativity research to study creativity in specific domains. However, the current state of the art of sports-related creativity research can be criticized in regards to empirical substantiation—as the hypothesis that improving players' DT leads to creative actions on the field is thus far untested—and additionally scrutinized in terms of the degree of theoretical elaboration—as creativity in team sports is currently not defined in terms of its functionality, but as an application of a historically rooted psychometric concept (i.e., Guilford, 1967). Alternatively, from a functional perspective, it has been suggested that the practical goal of developing creative players could be better approached without the detour of targeting a separate DT ability by directly targeting sensorimotor skills that allow players to solve situational tasks in many ways. Creativity, in turn, would not be the ability to be trained, but the result of situational skill training. By proposing an alternative point of view, the present paper aims to open a constructive discussion on the so far unchallenged assumptions of the predominant approach in the field and to encourage further empirical research on the topic.

## Data Availability Statement

The original contributions generated for the study are included in the article/Supplementary Material, further inquiries can be directed to the corresponding author/s.

## Author Contributions

All authors listed have made a substantial, direct and intellectual contribution to the work, and approved it for publication. Conception: SZ and E-JH; systematic literature search and analysis: SZ; writing and critical review: SZ and E-JH.

## Conflict of Interest

The authors declare that the research was conducted in the absence of any commercial or financial relationships that could be construed as a potential conflict of interest.
