# Supplementary material for: On the Issue of Developing Creative Players in Team Sports: A Systematic Review and Critique From a Functional Perspective
Source: Front Psychol. 2020 Oct 29;11:575475. doi: 10.3389/fpsyg.2020.575475 (PMC7658096; doi:10.3389/fpsyg.2020.575475)
Supplement: Supplementary file 1 [file Table_1.pdf]

Appendix A. PRISMA checklist

PRISMA 2009 Checklist

Section/topic	#	Checklist item	Reported on page #
**TITLE**			
Title	1	Identify the report as a systematic review, meta-analysis, or both.	1
**ABSTRACT**			
Structured summary	2	Provide a structured summary including, as applicable: background; objectives; data sources; study eligibility criteria, participants, and interventions; study appraisal and synthesis methods; results; limitations; conclusions and implications of key findings; systematic review registration number.	1
**INTRODUCTION**			
Rationale	3	Describe the rationale for the review in the context of what is already known.	2–3
Objectives	4	Provide an explicit statement of questions being addressed with reference to participants, interventions, comparisons, outcomes, and study design (PICOS).	3
**METHODS**			
Protocol and registration	5	Indicate if a review protocol exists, if and where it can be accessed (e.g., Web address), and, if available, provide registration information including registration number.	*
Eligibility criteria	6	Specify study characteristics (e.g., PICOS, length of follow-up) and report characteristics (e.g., years considered, language, publication status) used as criteria for eligibility, giving rationale.	3
Information sources	7	Describe all information sources (e.g., databases with dates of coverage, contact with study authors to identify additional studies) in the search and date last searched.	3
Search	8	Present full electronic search strategy for at least one database, including any limits used, such that it could be repeated.	3 / App. B
Study selection	9	State the process for selecting studies (i.e., screening, eligibility, included in systematic review, and, if applicable, included in the meta-analysis).	3–4** / Fig. 1
Data collection process	10	Describe method of data extraction from reports (e.g., piloted forms, independently, in duplicate) and any processes for obtaining and confirming data from investigators.	3–4
Data items	11	List and define all variables for which data were sought (e.g., PICOS, funding sources) and any assumptions and simplifications made.	4
Risk of bias in individual studies	12	Describe methods used for assessing risk of bias of individual studies (including specification of whether this was done at the study or outcome level), and how this information is to be used in any data synthesis.	***
Summary measures	13	State the principal summary measures (e.g., risk ratio, difference in means).	4****
Synthesis of results	14	Describe the methods of handling data and combining results of studies, if done, including measures of consistency (e.g., I^2) for each meta-analysis.	4****

PRISMA 2009 Checklist

Section/topic	#	Checklist item	Reported on page #
Risk of bias across studies	15	Specify any assessment of risk of bias that may affect the cumulative evidence (e.g., publication bias, selective reporting within studies).	***
Additional analyses	16	Describe methods of additional analyses (e.g., sensitivity or subgroup analyses, meta-regression), if done, indicating which were pre-specified.	-
**RESULTS**			
Study selection	17	Give numbers of studies screened, assessed for eligibility, and included in the review, with reasons for exclusions at each stage, ideally with a flow diagram.	3–4 / Fig. 1
Study characteristics	18	For each study, present characteristics for which data were extracted (e.g., study size, PICOS, follow-up period) and provide the citations.	Tab. 1
Risk of bias within studies	19	Present data on risk of bias of each study and, if available, any outcome level assessment (see item 12).	***
Results of individual studies	20	For all outcomes considered (benefits or harms), present, for each study: (a) simple summary data for each intervention group (b) effect estimates and confidence intervals, ideally with a forest plot.	Tab. 1
Synthesis of results	21	Present results of each meta-analysis done, including confidence intervals and measures of consistency.	4–14****
Risk of bias across studies	22	Present results of any assessment of risk of bias across studies (see Item 15).	***
Additional analysis	23	Give results of additional analyses, if done (e.g., sensitivity or subgroup analyses, meta-regression [see Item 16]).	-
**DISCUSSION**			
Summary of evidence	24	Summarize the main findings including the strength of evidence for each main outcome; consider their relevance to key groups (e.g., healthcare providers, users, and policy makers).	14
Limitations	25	Discuss limitations at study and outcome level (e.g., risk of bias), and at review-level (e.g., incomplete retrieval of identified research, reporting bias).	14
Conclusions	26	Provide a general interpretation of the results in the context of other evidence, and implications for future research.	14–16
**FUNDING**			
Funding	27	Describe sources of funding for the systematic review and other support (e.g., supply of data); role of funders for the systematic review.	-

Comments

*** Item 5, protocol and registration:** No protocol was preregistered. However, specific objectives of the review and methods were defined a priori:

(1) Objectives: To provide a complete overview of peer-reviewed research on the topic of creativity in team sport, with a main focus on disentangling different lines of research in terms of underlying conceptualizations and operationalizations of creativity, in order to facilitate the derivation of well-grounded practical recommendations based on the sport-scientific literature.

(2) Methods: Based on the review's objective, (a) eligibility criteria (p. 3), (b) information sources (p. 3), (c) search strategy (p. 3) and (d) to be extracted data items (p. 4) were specified before conducting the review.

**** Item 9, study selection:** The process of study selection was conducted by the first author. Potentially ambiguous cases were discussed with the second author and resolved by consensus after referring to the inclusion and exclusion criteria.

***** Item 12 / 15 / 19, risk of bias in / across individual studies:** Only studies published in peer-reviewed journals were considered. Based on the current review's objective, no quality assessment of individual studies was sought. However, regarding critical appraisal, fundamental assumptions of identified approaches to creativity in teams sport were critically discussed in regards to empirical substantiation and theoretical elaboration (p. 14–16).

**** **Item 13 / 14 / 21, summary measures / synthesis of results:** Based on the current review's objective and the inherent diversity in methodologies and definitions of creativity used in the reviewed studies, a narrative approach to synthesise information was conducted. Aiming to provide a comprehensible categorization of studies in regard to their conceptualization of creativity, three main comparative elements were used: (a) focus on one of the four P's (Rhodes, 1961), as employed regularly in creativity research (e.g., Kaufman, Plucker, & Baer, 2008), (b) the context in which creativity is manifested and (c) the task used to measure creativity. Identified conceptualizations represent the main summary measure and are reported narratively in the result section.

Appendix B. Full electronic search strategy

Last search: 17th June, 2019, via Campus Network Uni Bern

PsycINFO (via OvidSP)
((creativ* OR divergent thinking) AND (sport* OR football OR soccer)).tw.
limit to (all journal AND english langague)

Pubmed
((creativ*[Title/Abstract] OR "divergent thinking"[Title/Abstract]) NOT ("creative commons"[Title/Abstract])) AND (sport*[Title/Abstract] OR football[Title/Abstract] OR soccer [Title/Abstract])

ScienceDirect
Title, abstract or author-specified keywords: (creative OR creativity OR "divergent thinking") AND (sports OR sport OR football OR soccer)
limit to (research articles)

Scopus
TITLE-ABS-KEY ((creativ* OR "divergent thinking" AND NOT "creative commons") AND (sport* OR soccer OR football)) AND (LIMIT-TO (DOCTYPE , "ar")) AND (LIMIT-TO (LANGUAGE , "English")) AND (LIMIT-TO (SRCTYPE , "j"))

SportDiscus (via EBSCOhost)
TI ((creativ* OR “divergent thinking”) AND (sport* OR football OR soccer)) OR AB ((creativ* OR “divergent thinking”) AND (sport* OR football OR soccer))
Limiters - Peer Reviewed; Language: English; Publication Type: Academic Journal

Web of Science
(TS = (((creativ* NOT "creativecommons")OR "divergent thinking") AND (sport* OR soccer OR football))) AND LANGUAGE: (English) AND DOCUMENT TYPES: (Article)
